# Informed choice and consent among women attending for breast screening in the UK: data from a qualitative study

**DOI:** 10.1186/bcr3762

**Published:** 2015-11-05

**Authors:** Patsy Whelehan, Andrew Evans, Gozde Ozakinci

**Affiliations:** 1 University of Dundee, Dundee, UK; 2 University of St Andrews, St Andrews, UK

## Introduction

While the concept of overdiagnosis can be difficult to understand, it has been shown that women wish to be informed about it. The latest breast screening information leaflet offers considerable detail about potential benefits and harms of screening, including overdiagnosis. However, it is unknown how much use women attending for screening make of the leaflet. We report qualitative findings on informed choice and consent within the UK breast screening programme.

## Methods

Participants were clients and mammographers from breast screening units in Scotland and London. Semi-structured, in-depth, individual interviews were conducted and thematic analysis performed.

## Results

Twenty-two clients were interviewed, aged 50−72: seven first-attenders and 15 subsequent, from a range of deprivation categories. Eighteen mammographer-participants included assistant, registered, and advanced practitioners, with a wide range of ages and lengths of experience. Most clients understood that screening aims to detect breast cancer early to improve the chances of survival. Several were aware of the possibility of false positive results and the risk of mammography inducing a cancer. Others could not name any risks of screening. Women had mostly either skimmed the information leaflet or not read it at all. Several mammographers recounted experiences where women had appeared to attend under pressure from others and where severe challenges existed in ascertaining consent.

## Conclusion

These qualitative findings that some women attend for breast screening with little knowledge of the balance of risks and benefits, and in some cases may encounter coercion, require further investigation. New methods of communication may be indicated.

